# Effect of Voluntary Ethanol Consumption Combined with Testosterone Treatment on Cardiovascular Function in Rats: Influence of Exercise Training

**DOI:** 10.1371/journal.pone.0146974

**Published:** 2016-01-13

**Authors:** Sheila A. Engi, Cleopatra S. Planeta, Carlos C. Crestani

**Affiliations:** 1 Laboratory of Pharmacology, School of Pharmaceutical Sciences, Univ. Estadual Paulista-UNESP, Araraquara, SP, Brazil; 2 Joint UFSCar-UNESP Graduate Program in Physiological Sciences, São Carlos, SP, Brazil; University of São Paulo, BRAZIL

## Abstract

This study evaluated the effects of voluntary ethanol consumption combined with testosterone treatment on cardiovascular function in rats. Moreover, we investigated the influence of exercise training on these effects. To this end, male rats were submitted to low-intensity training on a treadmill or kept sedentary while concurrently being treated with ethanol for 6 weeks. For voluntary ethanol intake, rats were given access to two bottles, one containing ethanol and other containing water, three 24-hour sessions per week. In the last two weeks (weeks 5 and 6), animals underwent testosterone treatment concurrently with exercise training and exposure to ethanol. Ethanol consumption was not affected by either testosterone treatment or exercise training. Also, drug treatments did not influence the treadmill performance improvement evoked by training. However, testosterone alone, but not in combination with ethanol, reduced resting heart rate. Moreover, combined treatment with testosterone and ethanol reduced the pressor response to the selective α_1_-adrenoceptor agonist phenylephrine. Treatment with either testosterone or ethanol alone also affected baroreflex activity and enhanced depressor response to acetylcholine, but these effects were inhibited when drugs were coadministrated. Exercise training restored most cardiovascular effects evoked by drug treatments. Furthermore, both drugs administrated alone increased pressor response to phenylephrine in trained animals. Also, drug treatments inhibited the beneficial effects of training on baroreflex function. In conclusion, the present results suggest a potential interaction between toxic effects of testosterone and ethanol on cardiovascular function. Data also indicate that exercise training is an important factor influencing the effects of these substances.

## Introduction

Mental and substance use disorders are among major contributors to the burden of disease in the world [1]. Excessive ethanol consumption is the most prevalent condition among substance use disorders [1,2]. Cardiovascular dysfunctions constitute important complications associated with heavy ethanol use [3,4]. Indeed, several harmful cardiovascular effects have been reported following excessive ethanol consumption, including hypertension, cardiomyopathy, arrhythmia, coronary heart disease, and atherosclerosis [5,6]. Clinical and preclinical studies have demonstrated that alterations in contractile/relaxant properties of the vascular smooth muscle, changes in neuroendocrine function, impairment of baroreflex activity, and autonomic unbalance constitute important mechanisms underlying the negative cardiovascular effects of heavy ethanol consumption [3,4,6–8].

Abuse of androgenic—anabolic steroids (AASs) is also a serious public health problem [9,10]. For instance, clinical and preclinical studies have associated chronic AAS abuse with several cardiovascular dysfunctions, including hypertension, atherosclerosis, cardiac pathologies, impairment of baroreflex function, and changes in vascular function [11–13]. Most importantly, emerging data indicated that AAS abuse is associated with use of other substances. In fact, clinical evidence indicated that abuse of androgenic—anabolic steroids (AASs) was positively associated with ethanol use and dependence [14–16]. These findings are corroborated by preclinical studies showing that AAS can affect voluntary ethanol consumption and ethanol preference [17–19]. Despite the evidence that AAS and ethanol are co-abused, the potential toxic effects of the concomitant use of these substances are unknown.

Exercise is an important factor associated with ethanol consumption and AAS abuse. Indeed, a positive relationship between physical activity level and ethanol consumption have been demonstrated in humans across all ages [20]. To date, the factors related to this association in humans is unclear, but some authors have proposed that it would be an aware process of seeking of the exercise as a compensate mechanism for the excessive calories consumed from drinking [21,22]. However, evidence from preclinical studies has demonstrated that exercise can influence ethanol consumption and preference [23–27], possibly due to training-induced neuroplasticity in reward pathways [24]. This association is relevant to ethanol-evoked cardiovascular dysfunctions since previous studies have reported that exercise training attenuates the hypertension induced by ethanol [28,29]. However, the mechanisms underlying the beneficial cardiovascular effects of exercise in ethanol-treated animals are poorly understood.

The association between AAS abuse and exercise practice is well known [30]. Nevertheless, there is a lack in the literature of studies that investigated the influence of training in AAS-evoked cardiovascular changes [31]. Moreover, there is no evidence of the effect of exercise training on cardiovascular effects following combined use of ethanol and AAS. Therefore, our purpose in the present study was to evaluate the effects of voluntary ethanol consumption and testosterone treatment alone or in combination on basal values of arterial pressure and heart rate (HR), baroreflex activity, and blood pressure response to vasoactive agents in rats. Moreover, we investigated the possible protective effect of exercise training on these effects.

## Materials and Methods

### Animals

Sixty-seven male Wistar rats weighing approximately 200 g (50-days-old) in the beginning of the experiments were used. Animals were obtained from the animal breeding facility of the São Paulo State University-UNESP (Botucatu-SP, Brazil) and were housed in plastic cages in a temperature-controlled room at 24°C in the Animal Facility of the Laboratory of Pharmacology-UNESP. They were kept under a 12:12 h light-dark cycle (lights on between 7:00h and 19:00h). Housing conditions and experimental procedures were carried out following protocols approved by the Ethical Committee for Use of Animal and Subjects of the School of Pharmaceutical Sciences/UNESP (approval# 18/2013), which complies with Brazilian and international guidelines for animal use and welfare.

### Treatments

Voluntary ethanol consumption was performed using the *intermittent-access to 20% ethanol 2-bottle-choice drinking paradigm*, adapted from Simms et al. [32]. This is a free-choice method useful to estimate voluntary and spontaneous intake, as the animal is not forced to drink the ethanol solution and can choose whether to drink ethanol as well as the amount ingested over the time of exposure [32,33].

Rats were individually housed throughout the experiment and were given free access to two bottles during ethanol supply, one containing ethanol and other containing water. During the first 5 days (adaptation period), ethanol concentration was progressively increased daily (2%, 4%, 8%, 12%, 16%, or 20% v/v). On the 8^th^ day, the intermittent access begin, thus, rats were given 24h access to one bottle containing 20% ethanol and one bottle of water three times a week (Monday, Wednesday, and Friday) during 5 weeks. To determinate the amount of ethanol consumed, the bottles were weighted before and after the 24h period of ethanol access. Values of ethanol consumed were normalized to body weight and consumption is presented as g/kg/24h. Rats had free access to standard laboratory food throughout the experiment.

Treatment with testosterone (10 mg/kg, subcutaneously) was realized daily for 14 consecutive days. The doses and treatment regimen of testosterone were based on our previous studies [13,34,35].

### Exercise training

All animals were familiarized with exercise on a rodent treadmill (AVS Projetos, São Carlos, SP, Brazil) for one week. During the familiarization period, animals ran daily on the treadmill at a speed of 0.3 km/h and 0% grade for 10 min. No electrical stimulation was used to induce them to run [36]. Then, animals underwent a progressive maximal exercise test, which consisted on treadmill running with 0.3 km/h of increment each 3 min until exhaustion [37]. After the first maximal exercise test, animals were randomly allocated in sedentary and trained (both groups possessed the same physical capacity before training onset). Trained groups underwent a low-intensity training (50–60% of maximal exercise capacity, 0% grade) on the treadmill 1 h/day, 5 days/week for 6 weeks [37]. The sedentary groups were submitted once per week to a short period of mild exercise (10 min, 0.5 km/h, 0% grade) to keep them familiarized with treadmill environment and experimental procedures. Progressive maximal running test was repeated at weeks 4 and 6 in order to adjust training intensity and evaluate the efficacy of training protocol by comparing maximal capacity of sedentary and trained groups.

### Surgical Preparation

Animals were anesthetized with tribromoethanol (250 mg/kg, i.p.) and a catheter was inserted into the abdominal aorta through the femoral artery for cardiovascular recording. A second catheter was implanted into the femoral vein for the infusion of drugs. Both catheters were tunneled under the skin and exteriorized on the animal's dorsum. The catheters were filled with a solution of heparin (50 UI/ml, Hepamax-S^®^, Blausiegel, Cotia, SP, Brazil) diluted in saline (0.9% NaCl). After the surgery, rats were treated with a poly-antibiotic formulation with streptomycins and penicillins (560 mg/ml/kg, i.m.) to prevent infection and the non-steroidal anti-inflammatory drug flunixine meglumine (0.5 mg/ml/kg, s.c.) for postoperative analgesia.

### Measurement of Cardiovascular Parameters

The arterial cannula was connected to a pressure transducer (DPT100, Utah Medical Products Inc., Midvale, UT, USA). Pulsatile arterial pressure was recorded using an amplifier (Quad Bridge Amp, ML224, ADInstruments, NSW, Australia) and an acquisition board (PowerLab 4/30, ML866/P, ADInstruments, NSW, Australia). Mean (MAP), systolic (SAP), and diastolic (DAP) arterial pressure and HR values were derived from pulsatile arterial pressure recordings.

### Infusion of vasoactive agents

Intravenous infusion of the α_1_-adrenoceptor agonist phenylephrine (70 μg/ml at 0.4 ml/min/kg), the nitric oxide donor sodium nitroprusside (SNP) (100 μg/ml at 0.8 ml/min/kg), and acetylcholine (10 μg/ml at 1.2 ml/min/kg) was performed using an infusion pump (K.D. Scientific, Holliston, MA, USA) [8,13]. Phenylephrine caused incremental pressor effect while SNP and acetylcholine evoked incremental depressor responses.

### Assessment of baroreflex activity

Paired values of MAP and HR changes evoked by phenylephrine and SNP infusion were plotted to generate sigmoid logistic functions. The logistic equation was as follows:

$$\text{HR}=\;{\text{P}}_{1}+\;\left({\text{P}}_{2}-\;{\text{P}}_{1}\right)/1+\text{exp}\left[\text{B}{\text{P}}_{50}-\text{MAP}/\text{slope}\right]$$

Where P_1_ = lower HR plateau (bpm) (i.e., maximum reflex bradycardia), P_2_ = upper HR plateau (bpm) (i.e., maximum reflex tachycardia), P_2_ − P_1_ = HR range (bpm), slope = the steepness of the curve, BP_50_ = the MAP at 50% of the HR range [38]. The average gain (G, bpm/mmHg) is the average slope of the curves between +1 and -1 standard derivations from BP_50_ [38].

### Dose-response arterial pressure curves

The graded changes in MAP evoked by intravenous infusion of phenylephrine, SNP, and acetylcholine were plotted to generate dose—response curves [8,13]. Dose—effect curves were generated for each vasoactive agent by calculating the amount of drug infused and the MAP change each 2 s after starting the infusion. The maximal effect (E_max_) and the dose at 50% of the MAP range (ED_50_) for each vasoactive agent were compared in all experimental groups.

### Drugs

Phenylephrine hydrochloride (Sigma-Aldrich, St. Louis, MO, USA), sodium nitroprusside (Sigma-Aldrich), acetylcholine (Sigma-Aldrich) and tribromoethanol (Sigma-Aldrich) were dissolved in saline (0.9% NaCl). Ethanol (Labsynth, Diadema, SP, Brazil) was diluted in the drinking water. Testosterone propionate (PharmaNostra, Rio de Janeiro, RJ, Brazil) was dissolved in almond oil. Flunixine meglumine (Banamine^®^, Schering-Plough, Cotia, SP, Brazil) and the poly-antibiotic preparation (Pentabiotico^®^, Fort-Dodge, Brazil) were used as provided.

### Experimental procedures

**Fig 1:**
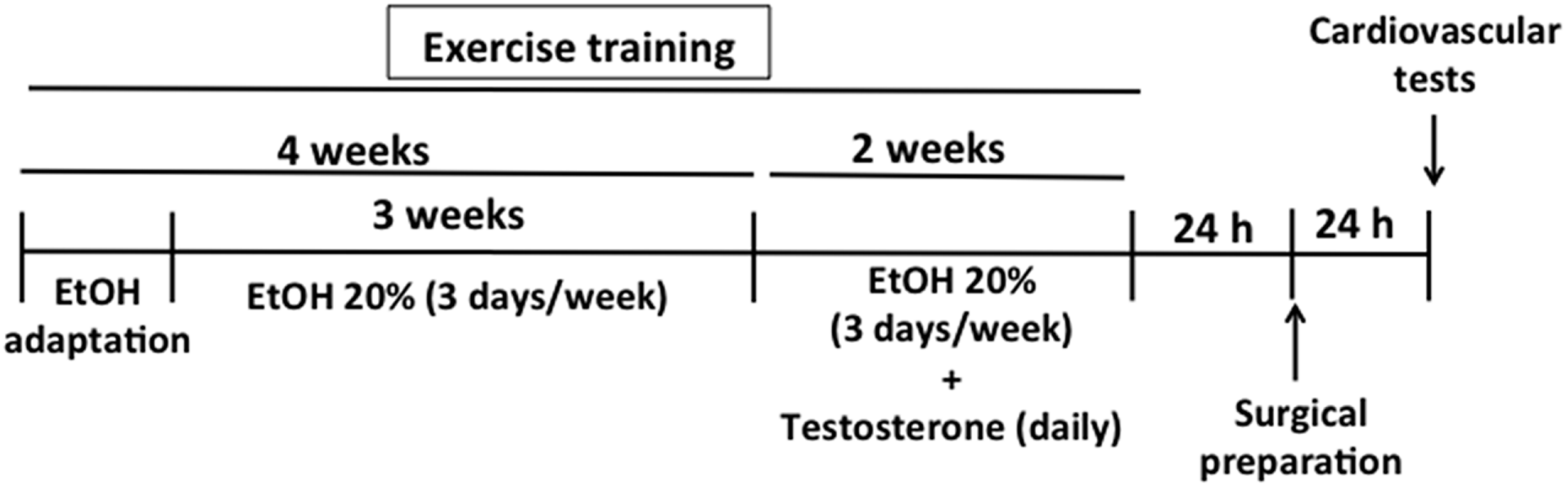
Schematic representation of the experimental protocol. Exercise training on the treadmill and ethanol treatment started on the same day and were realized for 6 weeks. During the first week (adaptation period), animals had continuous free access to two bottles, one containing ethanol and other containing water, and ethanol concentration was progressively increased daily until reach 20%. After this period, rats were given 24h concurrent access to one bottle containing 20% ethanol and other containing water three times a week (Monday, Wednesday, and Friday). In the last two weeks, animals underwent testosterone treatment concurrently with ethanol treatment and exercise training. Twenty-four hours after treatments and exercise training completion, animals in all experimental groups were subjected to surgical preparation, and the cardiovascular tests were performed 24 hours later. Rats had *ad libitum* food and water access throughout experimentation. EtOH—ethanol.

Different set of sedentary and trained animals were randomly allocated in four experimental groups: (i) control group (veh+veh), which animals were treated with almond oil (vehicle of testosterone, 1 ml/kg, s.c.) and the vehicle of ethanol (water, v.o.) (sedentary: n = 8, trained: n = 9); (ii) testosterone group (T+veh), which animals were treated with testosterone (10 mg/kg, s.c.) and the vehicle of ethanol (sedentary: n = 9, trained: n = 8); (iii) ethanol group (veh+EtOH), which consumed ethanol (20% *v/v*, drinking water) and were treated with almond oil (sedentary: n = 9, trained: n = 9); and (iv) testosterone + ethanol group (T+EtOH), which consumed ethanol and were treated with testosterone (sedentary: n = 7, trained: n = 8). Exercise training on the treadmill and ethanol treatment started on the same day and were realized for 6 weeks. For voluntary ethanol consumption, during all period of ethanol supply animals were given free access to two bottles, one containing ethanol and other containing water. During the first week, ethanol concentration was progressively increased daily until reach 20%. After this period, rats were given 24 h access to one bottle containing 20% ethanol and one bottle of water three times a week (Monday, Wednesday, and Friday). In the last two weeks (weeks 5 and 6), animals underwent testosterone treatment concurrently with ethanol treatment and exercise training. Protocols of treatment were based on our previous studies demonstrating cardiovascular changes following 10 days of daily administration of testosterone, whereas alterations in autonomic activity and cardiovascular function evoked by ethanol are mainly observed after 4 weeks of treatment [8,13,34,35]. Twenty-four hours after drug treatments and exercise training completion, animals in all experimental groups were subjected to surgical preparation, and the cardiovascular tests were performed 24 hours later. A schematic representation of the complete experimental protocol is presented in Fig 1.

On cardiovascular test day, animals were transferred to the experimental room in their home box and allowed 60 min to adapt to experimental room conditions, such as sound and illumination, before starting experiments. In the sequence, animals were subjected to a 30-min period of basal cardiovascular recording. After that, they received intravenous infusion of phenylephrine, SNP, and acetylcholine in a random order.

### Data Analysis

Data were expressed as mean ± SEM. All analysis of cardiovascular function were realized using two-way ANOVA, with treatment (testosterone and/or ethanol) and exercise (sedentary vs trained) as independent factors. Ethanol consumption and treadmill performance were analyzed using three-way ANOVA, with treatment and exercise as main independent factors and time as repeated measurement. When interactions between the factors were observed in two- and three-way ANOVA, groups were compared using Bonferroni’s *post hoc* test. Results of statistical tests with P<0.05 were considered significant.

## Results

### Effects of ethanol and/or testosterone treatment and training on treadmill performance

**Fig 2:**
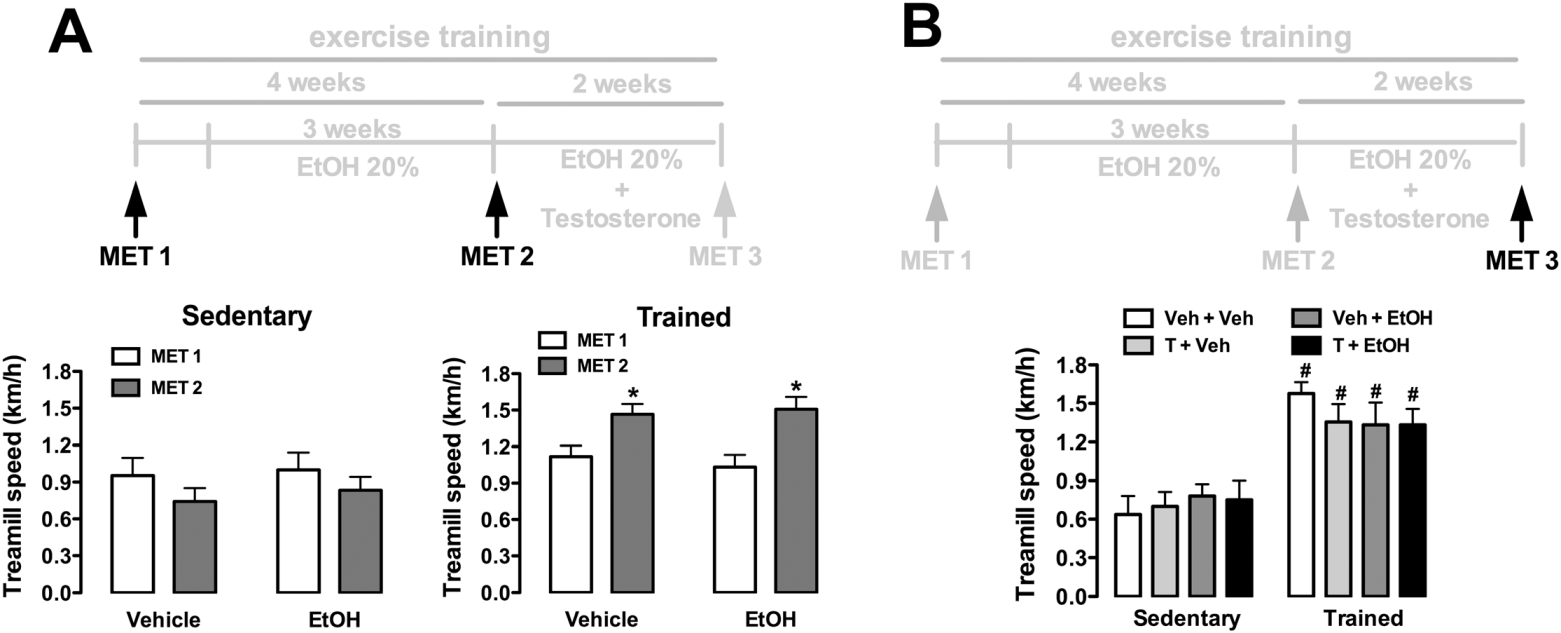
Maximal running speed (km/h) in maximal exercise tests (MET) in animals sedentary and subjected to exercise training on the treadmill (trained) treated with ethanol (EtOH) and/or testosterone (T). **(A)** Treadmill performance before the onset of T treatment in sedentary and trained animals treated with vehicle (water) or EtOH. The bars represent the mean±SEM. *P<0.05 vs MET1. Three-way ANOVA followed by Bonferroni’s *post hoc* test. (n = 16-18/group). **(B)** Treadmill performance after completion of treatments and exercise training protocol in animals sedentary and trained treated with EtOH and/or T. The bars represent the mean±SEM. #P<0.05 vs respective group sedentary. Two-way ANOVA followed by Bonferroni’s *post hoc* test. (n = 8-10/group).

Analysis of maximal running speed (km/h) in maximal exercise tests before the onset of testosterone treatment indicated a main effect of training (F_(1,65)_ = 17, P<0.0001), but without influence of ethanol consumption (F_(1,65)_ = 0.04, P>0.05) and time (F_(1,65)_ = 0.1, P>0.05) (Fig 2A). Analysis also indicated a training x time interaction (F_(1,65)_ = 8, P<0.006), but not training x treatment (F_(1,65)_ = 0.3, P>0.05) or treatment x time (F_(1,65)_ = 0.4, P>0.05) interactions. Analysis of treadmill performance after completion of drug treatments (ethanol and testosterone treatments) and exercise training indicated effect of training (F_(1,63)_ = 55, P<0.0001), but without influence of treatments (F_(3,63)_ = 0.1, P>0.05) and treatment x training interaction (F_(3,63)_ = 1, P>0.05) (Fig 2B).

### Effects of exercise training and/or testosterone treatment on ethanol consumption

**Fig 3:**
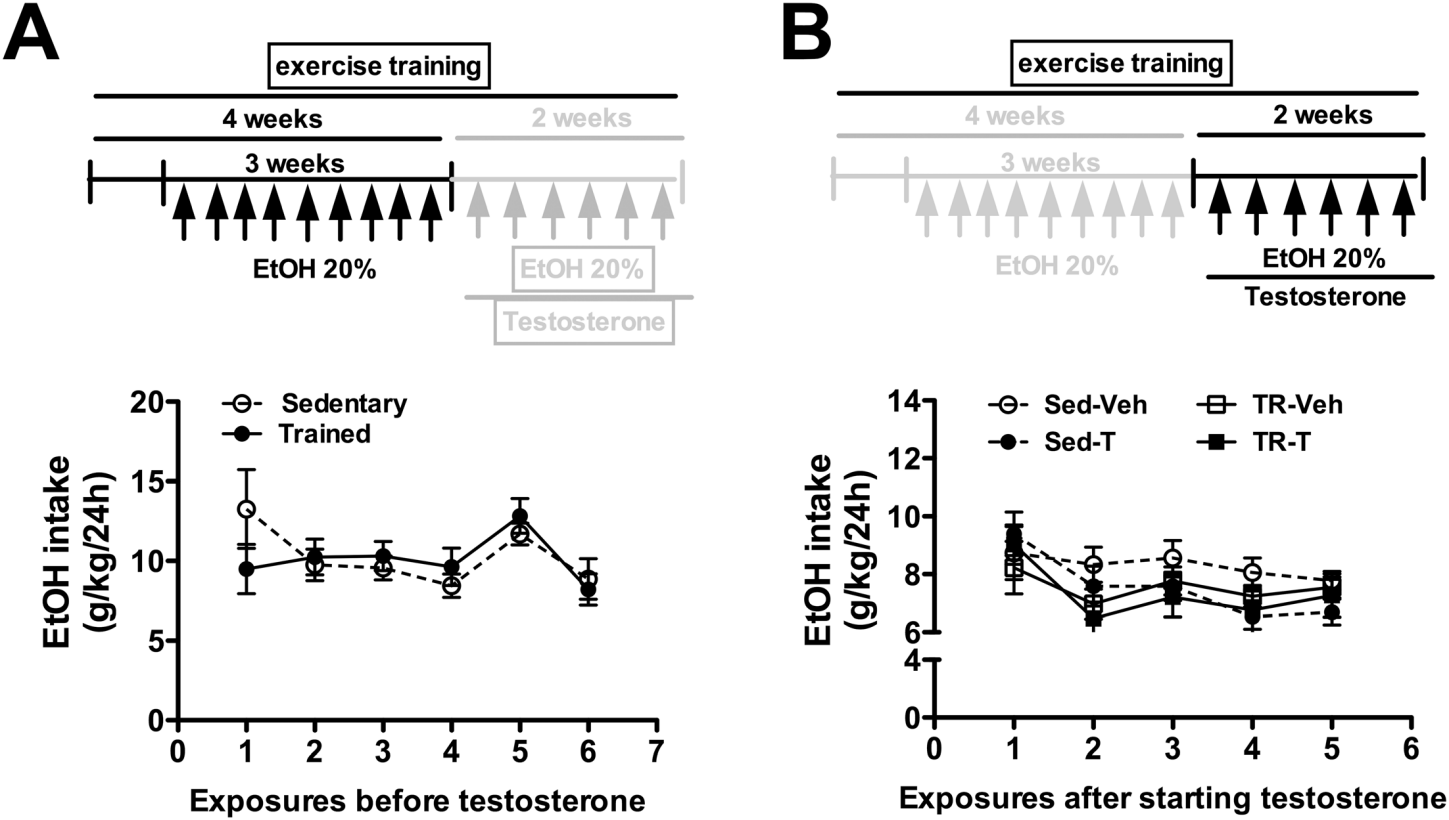
Voluntary ethanol intake (EtOH intake, g/kg/24h) in animals sedentary and subjected to exercise training on the treadmill (trained) treated with vehicle or testosterone (T). **(A)** Ethanol consumption before the onset of T treatment in sedentary and trained animals. The circles represent the mean±SEM. Two-way ANOVA followed by Bonferroni’s *post hoc* test (n = 16-18/group). **(B)** EtOH intake during treatment with vehicle or T in animals sedentary and trained. The circles represent the mean±SEM. Three-way ANOVA followed by Bonferroni’s *post hoc* test. (n = 8-10/group).

Analysis of ethanol intake before the onset of testosterone treatment indicated an effect over time (F_(5,202)_ = 3, P<0.01), but without a significant effect of exercise training (F_(1,202)_ = 0.03, P>0.05) and training x time interaction (F_(5,202)_ = 1, P>0.05) (Fig 3A). Comparisons of ethanol consumption during testosterone treatment indicated an effect over time (F_(4,128)_ = 8, P<0.0001), but without a significant effect of either exercise training (F_(1,32)_ = 1, P>0.05) or testosterone treatment (F_(1,32)_ = 1, P>0.05) (Fig 3B)

### Effects of ethanol and/or testosterone treatment and exercise training in arterial pressure and hear rate

**Fig 4:**
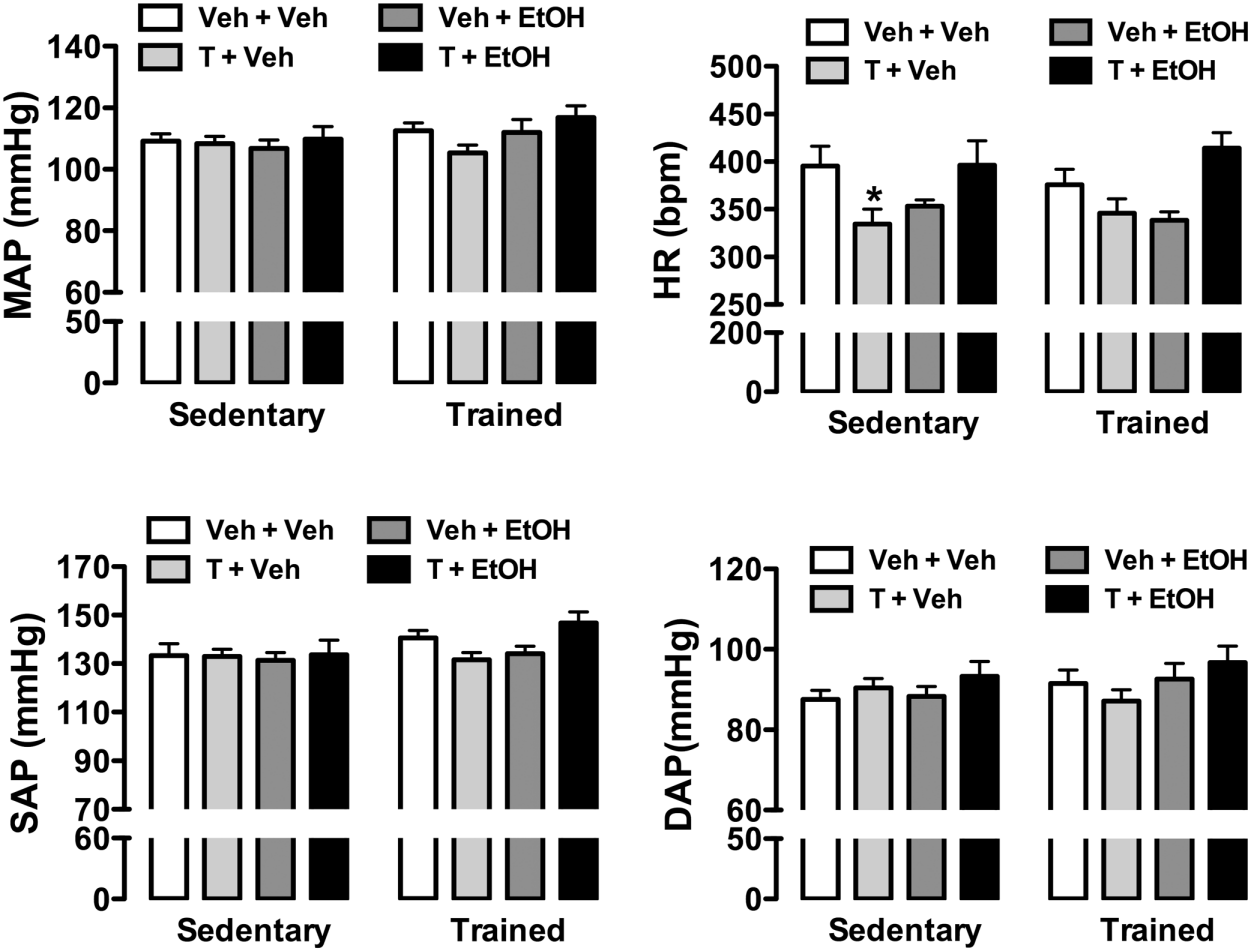
Mean (MAP), systolic (SAP), and diastolic (DAP) arterial pressure; and heart rate (HR) in animals sedentary and subjected to exercise training on the treadmill (trained) treated with ethanol (EtOH) and/or testosterone (T). The bars represent the mean±SEM. *P<0.05 vs respective group Veh+Veh within same condition. Two-way ANOVA followed by Bonferroni’s *post hoc* test. (n = 8-9/group).

Analysis of both MAP, SAP, and DAP indicated no effect of either drug treatments (MAP: F_(3,59)_ = 1, P>0.05; SAP: F_(3,59)_ = 2, P>0.05; DAP: F_(3,59)_ = 1, P>0.05) or exercise training (MAP: F_(1,59)_ = 2, P>0.05; SAP: F_(1,59)_ = 3, P>0.05; DAP: F_(1,59)_ = 0.8, P>0.05) (Fig 4). However, analysis of HR indicated a main effect of drug treatments (F_(3,59)_ = 8, P<0.0003), but without any influence of exercise training (F_(1,59)_ = 0.01, P>0.05) and treatment x training interaction (F_(3,59)_ = 0.6, P>0.05) (Fig 4). *Post-hoc* analysis revealed that testosterone treatment alone, but not in combination with ethanol (P>0.05), reduced HR in sedentary animals (P<0.05). This effect was not identified in trained rats (P>0.05) (Fig 4).

### Effects of ethanol and/or testosterone treatment and exercise training on baroreflex activity

**Fig 5:**
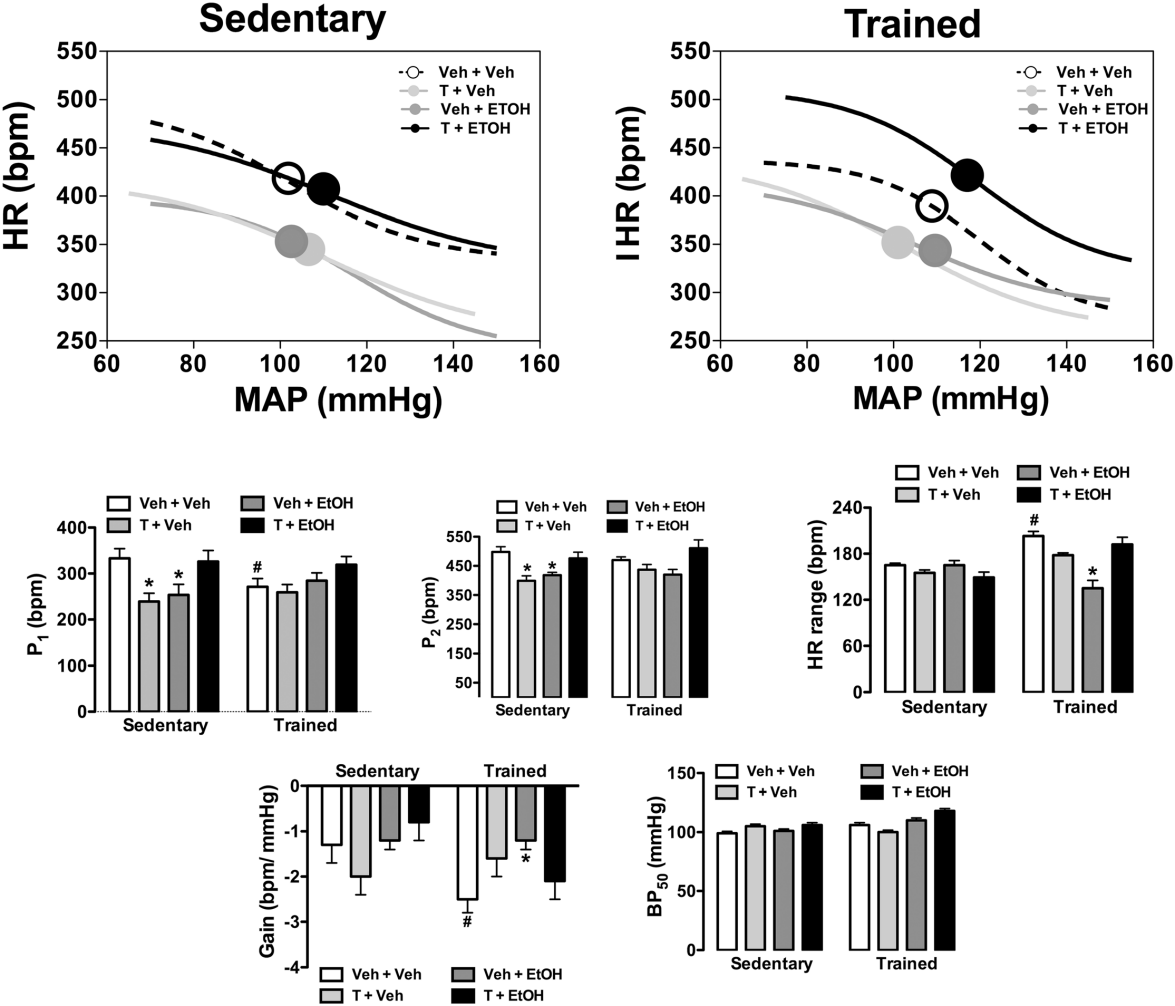
Analysis of baroreflex activity in animals sedentary and subjected to exercise training on the treadmill (trained) treated with ethanol (EtOH) and/or testosterone (T). **(Top)** Non-linear regression analysis of the baroreflex correlating mean arterial pressure change (MAP) evoked by intravenous infusion of phenylephrine and SNP and the reflex HR response (HR) in sedentary and trained animals treated with ethanol (EtOH) and/or testosterone (T). Symbols on sigmoid curves indicate the BP_50_. **(Bottom)** Parameters derived from nonlinear regression analysis of the baroreflex in sedentary and trained animals treated with ethanol (EtOH) and/or testosterone (T). The bars represent the mean±SEM. *P<0.05 vs respective group Veh+Veh within same condition, #P<0.05 vs respective group sedentary. Two-way ANOVA followed by Bonferroni’s *post hoc* test. (n = 8-9/group).

Results of the analysis of baroreflex activity are presented in Fig 5. The analysis indicated significant influence of drug treatments (HR range: F_(3,59)_ = 10, P<0.0001; BP_50_: F_(3,59)_ = 11, P<0.0001) and exercise training (HR range: F_(1,59)_ = 17, P<0.001; BP_50_: F_(1,59)_ = 17, P<0.0001) as well as a treatment x training interaction (HR range: F_(3,59)_ = 14, P<0.0001; BP_50_: F_(3,59)_ = 8, P<0.0002) for HR range and BP_50_ parameters. Analysis of P_1_ and P_2_ indicated a main effect of drug treatments (P_1_: F_(3,59)_ = 5, P<0.002; P_2_: F_(3,59)_ = 10, P<0.0001), but without influence of exercise (P_1_: F_(1,59)_ = 0.1, P>0.05; P_2_: F_(1,59)_ = 0.8, P>0.05) and treatment x training interaction (P_1_: F_(3,59)_ = 2, P>0.05; P_2_: F_(3,59)_ = 1, P>0.05). Analysis of the G indicated a main effect of exercise training (F_(1,59)_ = 5, P<0.03) and a treatment x training interaction (F_(3,59)_ = 3, P<0.03), but without influence of drug treatments (F_(3,59)_ = 2, P>0.05). *Post-hoc* analysis revealed that exercise training reduced P_1_ (P<0.05) and increased G (P<0.05) and HR range (P<0.05), and these effects were not observed in animals treated with testosterone and/or ethanol (P>0.05). Treatment with either testosterone or ethanol reduced both P_1_ (P<0.05) and P_2_ (P<0.05) in sedentary animals, but these effects were not identified in animals subjected to combined treatment with these substances (P>0.05). Furthermore, exercise training restored all changes on baroreflex function evoked by either testosterone treatment or voluntary ethanol consumption.

### Effects of ethanol and/or testosterone treatments and exercise training in arterial pressure changes evoked by vasoactive agents

**Fig 6:**
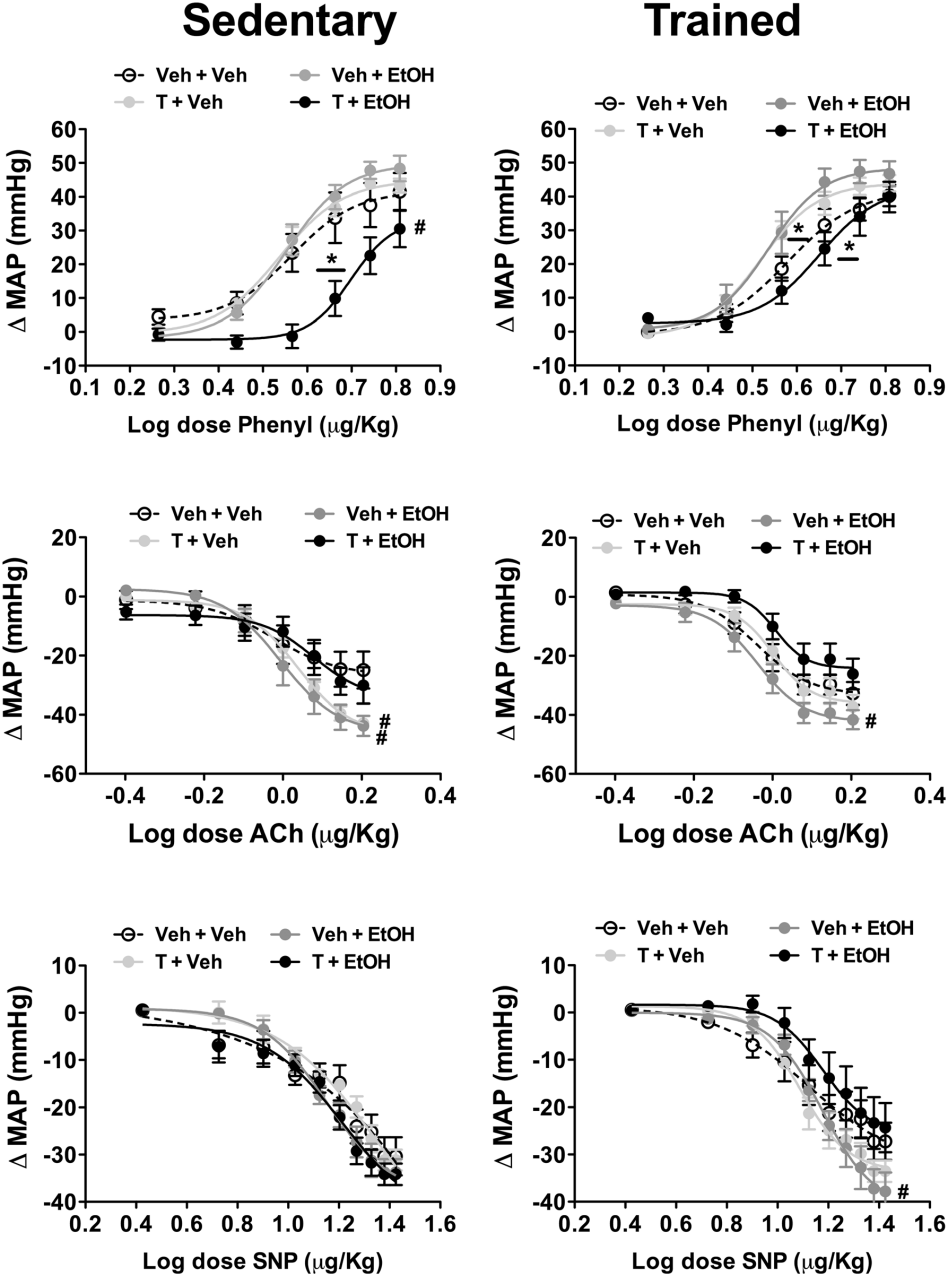
Mean arterial pressure change (ΔMAP) evoked by increasing concentrations of phenylephrine (Phenyl, top), acetylcholine (Ach, middle), and sodium nitroprusside (SNP, bottom) in in animals sedentary and subjected to exercise training on the treadmill (trained) treated with ethanol (EtOH) and/or testosterone (T). The circles represent the mean±SEM. *P<0.05 vs respective Veh + Veh group within same condition for ED_50_, ^#^ P<0.05 vs respective Veh + Veh group within same condition for E_max_. Nonlinear regression analysis. (n = 8-9/group).

Results of vascular reactivity to vasoactive agents are presented in Fig 6 and Table 1.

**Table 1:** Maximal effect (E_max_) and dose at 50% of the MAP range (ED_50_) for phenylephrine (Phenyl), acetylcholine (Ach) and sodium nitroprusside (SNP) dose-response curves in animals sedentary and subjected to exercise training on the treadmill (trained) treated with ethanol (EtOH) and/or testosterone (T).

Group	Phenyl		Ach		SNP	
	ED_50_	E_max_	ED_50_	E_max_	ED_50_	E_max_
**Sedentary**						
Veh + Veh	0.54±0.02	42±5	-0.03±0.04	-24±5	1.10±0.05	-31±4
T + Veh	0.53±0.007	43±2	0.02±0.005	-42±2*	1.17±0.02	-31±2
Veh + EtOH	0.54±0.008	49±3	-0.01±0.01	-43±3*	1.12±0.02	-33±2
T + EtOH	0.70±0.01*	29±4*	0.03±0.02	-30±5	1.11±0.01	-35±2
**Trained**						
Veh + Veh	0.57±0.01	40±3	-0.04±0.01	-32±3	1.10±0.04	-28±3
T + Veh	0.52±0.007*	44±2	-0.01±0.01	-38±3	1.09±0.01	-33±2
Veh + EtOH	0.53±0.01*	46±3	-0.05±0.01	-43±3*	1.15±0.02	-38±3*
T + EtOH	0.62±0.01*^#^	40±4	0.005±0.01	-27±3	1.15±0.02	-24±4

Values are mean ± SEM

* *P*<0.05 vs respective Veh+Veh group within same condition

^#^ *P*<0.05 vs respective sedentary group. Two-way ANOVA followed by Bonferroni *post hoc* test.

#### Phenylephrine

Intravenous infusion of the selective α_1_-adrenoceptor agonist phenylephrine dose-dependently increased arterial pressure in all experimental groups. Analysis of the E_max_ of the dose-response curves indicated a main effect of drug treatments (F_(3,59)_ = 6, P<0.002), but without influence of exercise training (F_(1,59)_ = 0.5, P>0.05) and treatment x training interaction (F_(3,59)_ = 2, P>0.05). Comparison of ED_50_ values indicated a main effect of drug treatments (F_(3,59)_ = 45, P<0.0001) and a treatment x training interaction (F_(3,59)_ = 5, P<0.006), but without influence of the training (F_(1,59)_ = 2, P>0.05). *Post-hoc* analysis revealed that combined treatment with testosterone and ethanol reduced E_max_ (P<0.05) and increased ED_50_ (P<0.05) in sedentary animals. The effect in E_max_ (P>0.05), but not ED_50_ (P<0.05), was restored by exercise training. Moreover, treatment with either testosterone or ethanol reduced ED_50_ in trained rats (P<0.05).

#### Acetylcholine

Intravenous infusion of acetylcholine dose-dependently reduced arterial pressure in all groups. Comparison of the E_max_ indicated a main effect of drug treatments (F_(3,59)_ = 10, P<0.0001), but without influence of exercise (F_(1,59)_ = 0.01, P>0.05) and treatment x training interaction (F_(3,59)_ = 1, P>0.05). Analysis of the ED_50_ indicated a significant effect of drug treatments (F_(3,59)_ = 5, P<0.007) and training (F_(1,59)_ = 6, P<0.02), but without a treatment x training interaction (F_(3,59)_ = 0.2, P>0.05). *Post-hoc* analysis revealed that treatment with either testosterone (P<0.05) or ethanol (P<0.05) enhanced E_max_ in sedentary animals, whereas in trained animals only ethanol increased this parameter (P<0.05).

#### Sodium nitroprusside

Systemic administration of the nitric oxide donor SNP dose-dependently reduced arterial pressure in all groups. Analysis of the E_max_ of the dose-response curves indicated a treatment x training interaction (F_(3,59)_ = 3, P<0.04), but without influence of either drug treatments (F_(3,59)_ = 2, P>0.05) or training (F_(1,59)_ = 0.8, P>0.05). Comparison of the ED_50_ did not indicate a significant effect of either treatment (F_(3,59)_ = 0.6, P>0.05) or training (F_(3,59)_ = 0.01, P>0.05). *Post-hoc* analysis revealed that ethanol consumption increased E_max_ in trained animals (P<0.05).

## Discussion

Present findings provide the first evidence of the effect of voluntary ethanol consumption combined with testosterone treatment on cardiovascular function of treadmill-trained rats. The main findings in the present study are: (i) exercise training improved the treadmill performance as evaluated in maximal exercise test, but neither ethanol consumption nor testosterone treatment affected this effect; (ii) voluntary ethanol consumption was not affected by either exercise training or repeated testosterone administration; (iii) voluntary ethanol consumption did not affect basal parameters of arterial pressure and HR, while testosterone treatment evoked resting bradycardia, which was not observed in animals submitted to combined treatment with ethanol or subjected to training on the treadmill; (iv) both testosterone treatment and voluntary ethanol consumption increased baroreflex-mediated bradycardia while tachycardia to blood pressure decrease was reduced. However, these baroreflex changes were not identified when substances were coadministrated. Exercise training restored all alterations on baroreflex function; and (v) drug treatments affected vascular reactivity to vasoactive agents (see details below), which was influenced by exercise training.

Our findings are in line with previous data demonstrating an improvement in physical capacity following low-intensity training on treadmill [37,39]. Previous studies demonstrated that testosterone treatment increased the running wheel activity in hamsters and rats [40,41]. However, to the best of our knowledge, present study is the first evaluating the effect of testosterone on treadmill performance in rodents. Results in humans demonstrated that hemodynamic and metabolic responses during an acute session of exercise on treadmill were impaired in AAS users [42]. Moreover, preclinical studies demonstrated that the cardiovascular beneficial effects of the exercise training were impaired by AAS administration in mice [43,44]. Thus, cardiovascular and metabolic negative effects may buffer a possible positive influence in performance related to anabolic actions of testosterone, thus explaining present findings. Regarding the ethanol, our findings are in line with earlier studies reporting that ethanol intake did not affect running wheel activity in rodents [24,25]. Present findings also corroborate clinical evidence that history of ethanol consumption does not affect exercise performance on treadmill [45].

The free-choice oral ethanol self-administration methods present face and construct validity as a model of human alcohol consumption once animals can choose whether to drink alcohol as well as the amount ingested over the time of exposure [33]. Therefore, in the present study we utilized *the intermittent access to 20% ethanol 2-bottle-choice drinking paradigm* for ethanol treatment [32]. The intermittent access to ethanol induces robust and reproducible levels of high voluntarily ethanol consumption over a long period of time without the use of any initiation procedures (e.g., sucrose fading or food and water deprivation) [32]. In fact, we detected ethanol intake in the range of 8–10 g/kg/24h throughout the experimental protocol, which is similar to those observed in alcohol-preferring rat strains [46] and in studies using protocols in which solution containing ethanol was the only source of liquid [47–50]. Previous results indicated that blood ethanol concentration following 30 minutes of voluntary ethanol consumption using the *intermittent-access to 20% ethanol drinking paradigm* ranged from 4 to 93 mg/dl in Wistar rats, and values significantly correlated with the amount of ethanol consumed [32]. Neither exercise nor testosterone affected ethanol consumption in the present study. Earlier studies reported that wheel running exercise reduced voluntary ethanol intake [23–27]. However, wheel running exercise has significant rewarding properties, so it has been proposed that a reduction in ethanol intake would be related to a substitution of rewarding effects of ethanol by hedonic properties of wheel running [24–26]. Regarding the influence of testosterone, previous studies have reported effect of AAS treatment in ethanol intake [17,18]. However, these studies investigated ethanol consumption following either a single acute administration of AAS [17] or 1–3 weeks after completion of chronic AAS treatment [18]. Thus, differences in experimental protocol may explain the discrepancy.

Clinical and preclinical studies have demonstrated the development of hypertension following long-term ethanol consumption [3–6,51]. Most of the studies in animals evaluating the ethanol-evoked hypertension used models in which solutions containing high concentrations of ethanol were the only liquid source (e.g., [49,52–55]). In fact, to the best of our knowledge, present findings are the first investigating the impact of voluntary ethanol consumption on cardiovascular function in rodents. Therefore, differences in experimental procedures may explain the discrepancy between our findings and earlier studies. For instance, the ingestion of water through free access to a water bottle throughout the experimental procedure may buffer some ethanol effects, thus minimizing the cardiovascular consequences. Indeed, an increase in circulating vasopressin, possibly related to dehydration [56], has been implicated in ethanol-evoked hypertension in rodents [53]. A possible impact of voluntary ethanol consumption in circulating vasopressin and hematocrit deserve further investigations, but differences in impact of voluntary vs forced ethanol intake in these parameters may constitute possible mechanisms explaining the discrepancies. Moreover, contrary to continuous consumption in models of forced ethanol administration, in the present study ethanol treatment was intermittent, which may maximize the impact of behavioral and physiological compensatory mechanisms in ethanol-evoked hypertension.

The resting bradycardia following treatment with testosterone is in line with previous studies [11,13,57]. The mechanism underlying this effect remains unclear. However, steroids can cross the blood—brain barrier and expression of androgen receptors has been documented in brain areas regulating cardiovascular function [58]. Treatment with AAS has been related to an increase in sympathetic activity [59]. Thus, AAS-evoked bradycardia is likely mediated by an increase in cardiac parasympathetic tone rather than a reduction in sympathetic activity. Interestingly, ethanol consumption inhibited the resting bradycardia to testosterone, which may be related to a cardiac sympathoexcitation evoked by ethanol intake [60,61]. Exercise training abolished the testosterone-evoked bradycardia, which is in line with a recent report showing that the impairment of cardiac function evoked by AAS was completely restored by treadmill exercise training [62].

Exercise increased reflex bradycardia during blood pressure increase, which is in line with earlier evidence [63]. Previous studies demonstrated that arterial pressure and HR changes evoked by either testosterone or ethanol were followed by changes on baroreflex activity [3,13]. Accordingly, we observed that treatment with either ethanol or testosterone increased baroreflex-mediated bradycardia while tachycardia to blood pressure decrease was reduced. The baroreflex changes were not identified when substances were coadministrated, thus further supporting an interaction between testosterone and ethanol on cardiovascular function. The baroreflex changes in testosterone-treated animals seems not to be related to the resting bradycardia since, for example, testosterone reduced the lower HR plateau (P_1_) of baroreflex function by ~30% while basal HR was reduced by ~15%. Impairment of baroreflex activity has been implicated in the etiology and development of hypertension [64]. It was previously reported that forced ethanol intake impaired reflex bradycardia [8,54,65], and this effect has been implicated in ethanol-induced hypertension [8,50]. Therefore, discrepancy in effects on baroreflex function support the evidence of a different impact of voluntary vs forced ethanol intake on arterial pressure. Indeed, facilitation of baroreflex-mediated bradycardia may constitute a compensatory mechanism counteracting the emergence of hypertension induced by both voluntary ethanol consumption and testosterone treatment. Impairment of baroreflex activity is also associated with overactivity of the sympathetic tone [66]. Thus, baroreflex changes do not seem to explain the resting bradycardia in testosterone-treated animals.

Training on the treadmill inhibited all changes on baroreflex function evoked by testosterone and ethanol treatments. As stated above, increased reflex bradycardia seems to be an important response counteracting arterial pressure changes, so that inhibition of these effects by exercise may be interpreted as a negative effect. Testosterone and ethanol also inhibited the effects of exercise training on baroreflex function as evidenced by inhibition of exercise-evoked facilitation of reflex bradycardia and increase in gain of baroreflex function, thus indicating that these substances may affect the beneficial cardiovascular effects of exercise training. These results are in line with previous data demonstrating that AAS suppressed cardiovascular adaptation to training [43,44]. Exercise training attenuated the hypertension induced by forced ethanol consumption [28,29]. However, to the best of our knowledge, present findings provide the first evidence of the impact of exercise on baroreflex function of ethanol-treated animals.

Testosterone treatment and ethanol consumption in combination, but not alone, reduced the pressor response to phenylephrine. A vascular hyperreactivity to vasoconstrictor agents has been associated with hypertension [67], so that reduced responsivity to vasoconstrictor agents may be an important mechanism counteracting an increase on arterial pressure. The facilitation of depressor response to vasodilator agents following treatment with either testosterone or ethanol is in line with previous studies [8,13,54]. Our findings are also supported by evidence that testosterone acting directly in vascular wall induces relaxation of vascular smooth muscle [68]. Also, long-term ethanol consumption has been related to an inhibition of AChE activity [69,70], which can underlie the increased depressor response to acetylcholine in ethanol-treated animals. However, the absence of changes in pressor response to phenylephrine in ethanol-treated animals contrast with *in vitro* and *in vivo* studies reporting that forced ethanol consumption increased vascular reactivity to phenylephrine [4,8,50,54]. Nevertheless, these data are in line with arterial pressure results in which forced, but not voluntary, ethanol intake evoked hypertension.

Exercise training inhibited the reduction of pressor response to phenylephrine following combined treatment with testosterone and ethanol as well as the facilitation of depressor response to acetylcholine in testosterone-treated animals. These results contrast with the well-documented effects of exercise in increasing vascular nitric oxide availability and reducing vascular reactivity to α-adrenoceptor agonists [71]. A possible mechanism underlying the protector effect of training in these alterations on vascular reactivity can be an influence of exercise in pharmacokinetic of testosterone and ethanol, which in turn may affect the circulating levels of these substances. For instance, exercise training on treadmill can increase rates of ethanol clearance [72], while a decrease in metabolic clearance of testosterone was reported following an acute session of exercise [73]. The impact of training in testosterone clearance is less understood, but a study reported lower testosterone levels in runners vs sedentary subjects [74]. In addition, impairment in response of vascular relaxation to β-adrenoceptor agonist was reported following exercise training on treadmill [75]. Vasoconstrictor response to phenylephrine is counteracted by β_2_-adrenoceptor [76]. Thus, reduction in vasorelaxation response of β-adrenoceptors may also account to exercise effect in inhibiting the influence of the combined treatment with testosterone and ethanol in phenylephrine response. This mechanism may also underlie the facilitation in vascular responsiveness to phenylephrine observed in trained animals treated with either testosterone or ethanol. Facilitation of depressor response to SNP in trained rats treated with ethanol is line with evidence that exercise training increases vascular smooth muscle sensitivity to nitric oxide [71].

In summary, although combined treatment with testosterone and ethanol did not affect baseline arterial pressure and HR parameters, important changes on cardiovascular function were identified, including reduction in pressor responsiveness to phenylephrine. Reduced vascular reactivity to vasoconstrictor agents may counteract other effects on cardiovascular function so inhibiting the emergence of changes in arterial pressure. Effects of treatment with testosterone and ethanol alone on baroreflex activity and depressor response to acetylcholine were inhibited when substances were coadministrated. These results provide evidence that these substances are capable of mutually inhibiting the cardiovascular effects of each other, thus further supporting an interaction between their toxic effects on cardiovascular function. Regarding the influence of exercise training, we observed that training on the treadmill inhibited cardiovascular effects of drug treatments, but some effects were identified only in trained animals. Taken together, these results indicate the exercise as an important factor affecting the effects of ethanol and testosterone on cardiovascular function. Indeed, present data suggest that cardiovascular effects of these substances may be related, at least in part, to an inhibition of protector effects of exercise training on cardiovascular function.
