# Correction: Effect of Voluntary Ethanol Consumption Combined with Testosterone Treatment on Cardiovascular Function in Rats: Influence of Exercise Training

**DOI:** 10.1371/journal.pone.0151749

**Published:** 2016-03-10

**Authors:** Sheila A. Engi, Cleopatra S. Planeta, Carlos C. Crestani

There is an error in affiliation 1 for all authors. Affiliation 1 should be: Faculdade de Ciências Farmacêuticas, UNESP—Univ Estadual Paulista, Araraquara, SP, Brasil.
